# Polymer Functional Layers for Perovskite Solar Cells

**DOI:** 10.3390/polym17192607

**Published:** 2025-09-26

**Authors:** Jinho Lee, Jaehyeok Kang, Jong-Hoon Lee, Soonil Hong

**Affiliations:** 1 Department of Physics, Incheon National University, Incheon 22012, Republic of Korea; 2 Department of Advanced Materials Engineering, Kyonggi University, Suwon 16227, Republic of Korea; rkdwogur2397@kyonggi.ac.kr; 3 Division of Advanced Materials, Korea Research Institute of Chemical Technology (KRICT), Daejeon 34114, Republic of Korea

**Keywords:** polymers, perovskite solar cells, functional layers

## Abstract

Perovskite solar cells (PSCs) are next-generation solar cells; they are replacing silicon-based solar cells due to their higher efficiency, greater cost-effectiveness, and enhanced potential for various applications. Exceeding the efficiency of crystalline silicon-based solar cells, the commercialization of PSCs has driven not only the development of perovskite photoactive materials but also charge transport layer advancements, interfacial engineering, and processing technologies. PSCs were developed later than dye-sensitized solar cells and organic solar cells; the adoption of techniques previously employed in these technologies is significant to enhancing their performance. Among them, polymers are widely employed in perovskite solar cells to facilitate efficient charge transport, provide interfacial passivation, enhance mechanical flexibility, enable solution-based processing, and improve environmental stability. In this review, we highlight the roles of polymer materials as charge transport layers, interfacial layers, and other functional layers for highly efficient and stable PSCs.

## 1. Introduction

Perovskite solar cells (PSCs) have attracted considerable attention as a renewable energy source due to their rapid increase in efficiency and potential for diverse applications [1,2,3,4]. Since their initial power conversion efficiency (PCE) of 3.8% in 2009, continuous developments in composition engineering of perovskite, charge transport materials, and interfacial engineering have enabled the PCE to reach 27% [1,2,3,4,5,6,7,8]. In the early stages, PSCs were developed on porous TiO_2_ scaffolds, originally used in dye-sensitized solar cells (DSSCs), with perovskite materials infiltrating the pores and serving as the light-absorbing layer [5,9,10,11]. In addition, dye-sensitized perovskite and liquid-type hole transport layers (HTLs) have been replaced with solid-type layers because solid-state solar cells have many advantages, such as elimination of the liquid electrolyte leakage issue, higher mechanical durability, and better scalability for large-area cells, making them more suitable for commercialization [9,10,11]. In this context, spiro-OMeTAD, originally used in light-emitting diodes and later adopted in solid-state DSSCs, started to be applied as the HTL in PSCs. N. G Park’s group first reported all-solid-state thin film PSCs using submicron thick mesoporous TiO_2_ with an infiltrated perovskite absorber and spiro-OMeTAD HTL [9].

Owing to their facile fabrication process, planar-type PSCs have been developed, and a wide range of materials originally used in dye-sensitized solar cells, light-emitting diodes, and OSCs began to be incorporated [11,12,13,14,15]. Alternatively, new materials such as metal oxides, organic molecules, and polymers are sometimes developed and applied to other solar cells first and then to PSCs, or vice versa [16,17,18,19]. From these efforts, both mesoporous n–i–p and planar p–i–n structures have achieved comparably high efficiencies, while extensive research is currently focused on enhancing their stability and enabling large-area scalability [8,13,20,21,22]. Most recently, a 20% exceedance of PCE on an area greater than 200 cm^2^ has been certified and listed in the National Renewable Energy Laboratory (NREL) chart [6,23]. However, high-temperature processes and spin-coating techniques, which are challenging to implement in commercial manufacturing and flexible applications, are still commonly employed [23,24,25,26,27]. Polymers have been utilized to enhance the performance of perovskite solar cells because of their compatibility with large-area fabrication processes, such as low-temperature and printing techniques, and their flexible nature [28,29,30].

In this review, we discuss in detail how polymers can be utilized as various functional layers, such as charge transport, interfacial, electrode encapsulation, and anti-reflection layers, in the construction of highly efficient and stable PSCs. The core challenges presented by polymers for functional layers in PSCs include the need for precise energy-level alignment, stable conductivity control, and reliable doping strategies, while maintaining film uniformity and large-area processability. We briefly discuss the fundamental principles and suitability of using various polymers as various functional layers. Our aim with this review is also to address the potential use of polymer functional layers in all perovskite tandem solar cells to achieve high efficiencies, as well as the necessary improvements required for their further development.

## 2. Polymers for PSCs

A PSC device structure consists of substrate/bottom electrode/electron transport layer (ETL) or hole transport layer (HTL)/Perovskite/HTL or ETL/top electrode [11]. A configuration of HTL/perovskite/ETL from the bottom is denoted as a p–i–n structure, while ETL/perovskite/HTL is denoted as an n–i–p structure. In the case of PSCs, the early development was based on mesoporous TiO_2_, leading to the initial advancement of n–i–p structured PSCs, which are referred to as the “normal” structure, while p–i–n structures were developed later and are thus called the “inverted” structure [9,10,11]. For high-efficiency n–i–p structured PSCs, metal oxide-based ETLs, such as SnO_2_ and TiO_2_, and small-molecule organic hole transport layers, like Spiro-OMeTAD, have been widely used [11]. With the development of p–i–n structures, polymeric materials such as PEDOT:PSS and PTAA began to be employed due to their high mobility and transparency in the visible light range [12]. In addition to these materials, various polymers are employed as functional layers to improve efficiency and stability and enable large-area fabrication.

**Figure 1:**
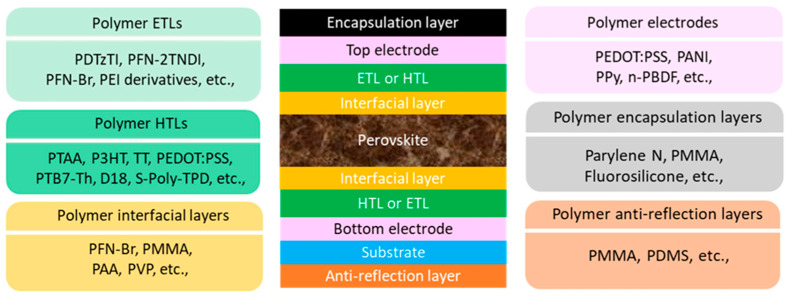
A schematic of PSC structure that includes representative polymer-based functional layers such as ETLs, HTLs, interfacial layers, electrodes, encapsulation layers, anti-reflection layers.

In OSCs, polymers have been widely employed to form entire layers including electrodes, HTLs, photoactive layers, ETLs, and interfacial layers [17,19]. In PSCs, except for cases where polymers are added as additives to the photoactive perovskite layer, they are generally used either as individual functional layers or in combination with other materials to form complexes [30,31,32]. Depending on their bandgap, conductivity, and energy levels, polymers are mainly utilized as ETLs or HTLs, and they can also be employed as electrodes, an encapsulation layer, anti-reflection layer, and interfacial layer, such as a passivation layer, as shown in Figure 1. In PSCs, polymer ETLs facilitate charge collection not primarily through high conductivity- based transport, but rather via dipole formation inducing vacuum level shifting, as typically observed with PFN-Br or PEI derivatives. Typical examples of HTLs are P3HT, PTAA and PEDOT:PSS, the HOMO level is between 5.0~5.2 eV, which is well aligned to the perovskite photoactive layer. The conductivity can be enhanced by the doping process; notably, the conductivity of PEDOT:PSS can be enhanced to values exceeding 1000 S cm^−1^ through appropriate doping, which has rendered it suitable for use as a transparent electrode. [10,11,33]. Moreover, polymers with high charge mobility and minimal absorption in the visible region can also be utilized as recombination layers in perovskite tandem solar cells [34,35,36,37,38].

Therefore, when selecting or engineering polymers for functional layers in PSCs, several molecular design principles are critically important. First, the energy level alignment, particularly the HOMO/LUMO positions, should match those of adjacent perovskite or transport layers to facilitate efficient charge extraction or blocking. Second, the side-chain polarity and functional groups must be tailored to enhance miscibility, film formation, and interfacial compatibility with the perovskite surface. Backbone rigidity and planarity influence charge mobility and film morphology, especially in conducting or semiconducting polymers. Additionally, optical bandgap engineering is essential when transparency is needed (e.g., in electrodes or interlayers), while chemical stability and hydrophobicity are crucial for long-term device durability. An integrated molecular design approach that balances electronic, morphological, and interfacial properties is thus indispensable for optimizing polymer performance across different PSC applications. In the following sections, the utilization of polymers as functional layers will be discussed based on previously reported research and development cases.

## 3. Polymers as ETLs for PSCs

**Figure 2:**
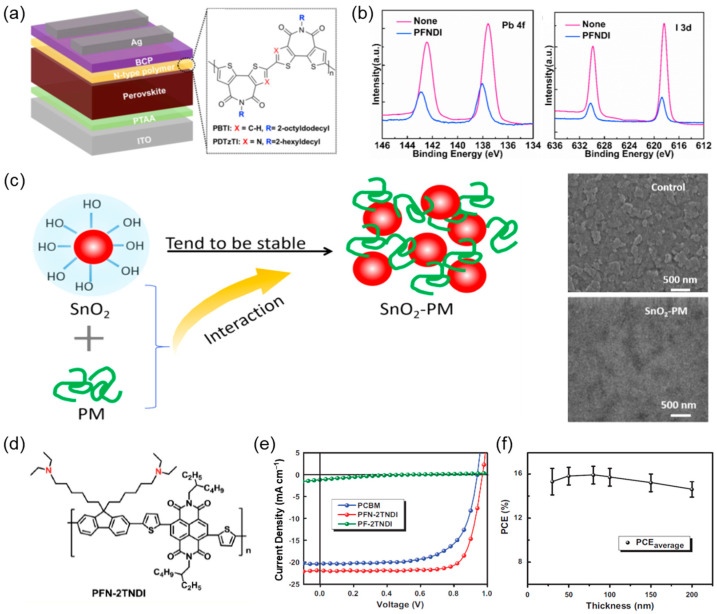
(**a**) The schematic diagram of the inverted planar perovskite solar cells with polymer electron transport layers and molecular structures of the n-type polymers PDTzTI and PBTI. Adapted with permission from Ref. [39] Copyright 2020, Elsevier. (**b**) XPS spectra of the perovskite films with and without PFNDI layer for (**left**) P 2p and (**right**) I 3d spectra. Adapted with permission from Ref. [40] Copyright 2020, Elsevier. (**c**) Schematic diagram of the SnO_2_ colloidal dispersion regulated by PM, and scanning electron microscopy (SEM) images of the SnO_2_ and SnO_2_-PM films. Adapted with permission from Ref. [42] Copyright 2022, Royal Society of Chemistry. (**d**) The chemical structure of PFN-2TNDI and (**e**) photovoltaic performance of PSCs with different ETLs, and (**f**) effects of the thickness of PFN-2TNDI ETL on the PCE of PSCs. Adapted with permission from Ref. [43] Copyright 2016, John Wiley and Sons.

Polymeric materials have emerged as promising alternatives to conventional fullerene-based ETLs in PSCs, offering distinct advantages in terms of energy level tunability, interfacial defect passivation, processability, and operational stability. In particular, the development of n-type conjugated polymers with tailored backbones and side chains has enabled their successful application as pure ETL materials, without blending with inorganic components. In the course of the initial demonstrations, a thiazole-imide-based conjugated polymer (PDTzTI) was demonstrated to function effectively as an ETL in inverted (p–i–n) architectures [39]. The material exhibited favorable alignment with the conduction band of the perovskite absorber and contributed to strong interfacial interactions through its sulfur and nitrogen atoms, which coordinated with undercoordinated Pb^2+^ ions. These features have been shown to reduce trap states and improve film morphology, resulting in a PCE of 20.86% and improved long-term stability, highlighting the potential of rational polymer design for ETL functionality (Figure 2a).

Further advancement was marked by the conception of PFNDI, a naphthalene diimide (NDI)-based polymer bearing phosphite ester side chains [40]. The molecular structure promoted solubility and enabled strong interfacial coordination, which facilitated the formation of uniform films and reduced trap-assisted recombination. Devices fabricated with PFNDI achieved efficiencies up to 18.25% and retained over 80% of their performance after prolonged exposure to ambient conditions. Spectroscopic analysis confirmed the presence of P–O–Pb interactions, thereby affirming the chemical basis of the observed passivation effect (as shown in Figure 2b). Another example involves PFN-2TNDI, an amino-functionalized copolymer containing fluorene, thiophene, and NDI units, which was implemented as an ETL in conventional (n–i–p) devices [41]. The incorporation of amino groups improved energy level alignment and enabled strong chemical passivation of undercoordinated lead ions. As a result, the PSCs displayed a PCE of 15.96% and excellent photostability under continuous 365 nm illumination, retaining 75% of their initial performance after 3000 h—significantly surpassing the UV durability of TiO_2_-based ETLs.

Polymer–inorganic hybrid strategies have also proven effective. In one such approach, dendritic poly(amidoamine) (PM) was incorporated into SnO_2_ colloids to create a polymer-complexed ETL [42]. The presence of PM prevented nanoparticle agglomeration, enhanced surface smoothness, and improved wettability. These morphological and interfacial enhancements facilitated the growth of high-quality perovskite films with reduced trap densities (Figure 2c). Devices utilizing such a hybrid ETL architecture reached a PCE of 22.93% with an open-circuit voltage (*V_oc_*) of 1.17 V. These devices exhibited remarkable stability under ambient conditions. Additionally, conjugated polymers based on polyfluorene and NDI frameworks, functionalized with amino groups, have been shown to suppress hysteresis and enhance open-circuit voltage by improving contact quality and energy alignment at the electron-collecting interface [43]. In comparison with metal oxide ETLs, these polymeric systems demonstrated superior light stability, retaining device performance under extended UV irradiation (Figure 2d–f).

Overall, these studies illustrate the versatility of polymer-based ETLs. Whether employed as pure semiconducting transport layers or integrated into hybrid systems with inorganic oxides, conjugated polymers offer a tunable platform for optimizing charge extraction, interface quality, and long-term stability. The compatibility of these materials with solution processing and low-temperature fabrication further supports their integration into flexible and scalable perovskite photovoltaic technologies.

## 4. Polymers as HTLs for PSCs

**Figure 3:**
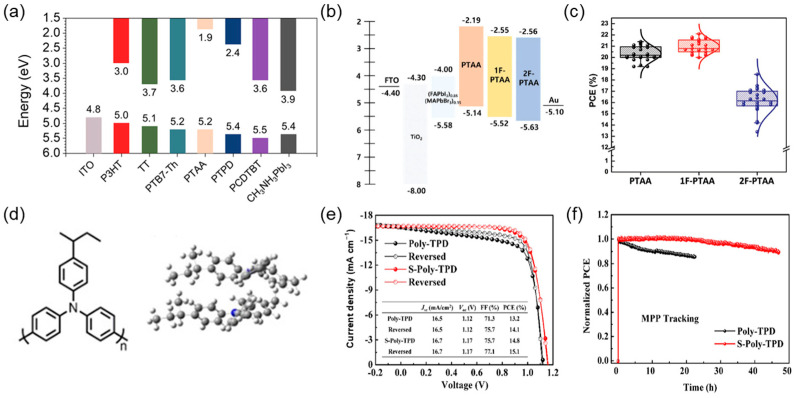
(**a**) Energy level diagram for perovskite (MAPbI_3_) and representative polymeric HTLs. (**b**) Energy diagrams of perovskite devices with PTAA derivatives as HTLs and (**c**) statistical data of PCE and *V_oc_* were obtained from the devices for each different HTL. Reprinted with permission from Ref. [47] Copyright 2018, John Wiley and Sons. (**d**) Molecular structure and density functional theory (DFT) calculation results of the stacking model of S-Poly-TPD, and (**e**) J–V characteristics and (**f**) maximum power point (MPP) tracking properties of PSCs. Adapted with permission from Ref. [49] Copyright 2022, John Wiley and Sons.

In the device structure of PSCs, polymeric HTLs are critical components that facilitate high efficiency and ensure long-term stability. Such excellent film-forming properties, suitable energy levels, and chemical resilience render them particularly attractive for both laboratory-scale and scalable device fabrication. Most notably, the inherent hydrophobicity of polymers assists in protecting the perovskite absorber from moisture, while their tunable electronic structure enables precise energy level alignment with the perovskite valence band, thereby minimizing interfacial recombination losses (Figure 3a). Among various candidates, poly[bis(4-phenyl)(2,4,6-trimethylphenyl)amine] (PTAA) has been identified as a benchmark polymer HTL, especially in inverted (p–i–n) device configurations. PTAA offers a deep highest occupied molecular orbital (HOMO) level that facilitates efficient hole collection and forms uniform, pinhole-free films that ensure intimate contact with the perovskite layer. In contrast to small-molecule HTLs such as Spiro-OMeTAD, PTAA exhibits higher conductivity without dopants, which enables its application not only in n-i-p structured PSCs but also in p-i-n structured PSCs [10,44,45,46]. As a result, PTAA-based PSCs exhibit superior operational stability, even under environmental stress, by preventing ion migration and moisture adsorption.

Although PTAA has been widely adopted, its relatively poor wettability and limited interfacial adhesion present persistent challenges, particularly on hydrophilic substrates or in large-area printing processes. Recent studies were aimed at addressing these limitations by chemically modifying PTAA or introducing functional additives. One such strategy involves the incorporation of fluorinated side chains into the PTAA backbone. This modification not only enhances hydrophobicity but also introduces interfacial dipole interactions that lower energetic disorder, improve hole mobility, and suppress trap-assisted recombination. As illustrated in Figure 3b,c, devices with fluorinated PTAA derivatives have consistently outperformed their unmodified counterparts in *V_oc_* and PCE metrics [47]. An alternative approach involves blending PTAA with insulating polymers such as poly(methyl methacrylate) (PMMA), which improves surface coverage and wetting behavior without compromising charge transport. The resulting composite HTLs exhibit improved film homogeneity and interface contact, which contribute to the stabilization of the perovskite morphology and the suppression of interfacial defects [48].

In addition to modifications to PTAA, new classes of triphenylamine-based polymers have been designed to offer both electronic compatibility and mechanical robustness. For instance, the application of side-chain engineering has proven effective in enhancing polymer packing and interfacial interactions. A notable example is the side-chain-modified triphenylamine polymer (S-Poly-TPD) developed by Xie et al., which replaces linear alkyl chains with branched isobutyl groups [49]. This ostensibly subtle change improves molecular stacking, enhances hole mobility, and reduces nonradiative losses at the perovskite/HTL interface. Devices employing S-Poly-TPD achieved a certified PCE of 21.3% and maintained over 90% of initial performance after prolonged operation under illumination (Figure 3d–f).

These diverse developments reflect a broader trend toward rational molecular engineering of polymeric HTLs, with the aim of addressing the key limitations of scalability, interfacial compatibility, and long-term operational stability. The evolution of polymer HTLs, through means such as fluorination, side-chain engineering, and anchoring strategies, is of critical importance in the context of next-generation PSC technologies.

## 5. Polymers as Interfacial Layers for PSCs

**Figure 4:**
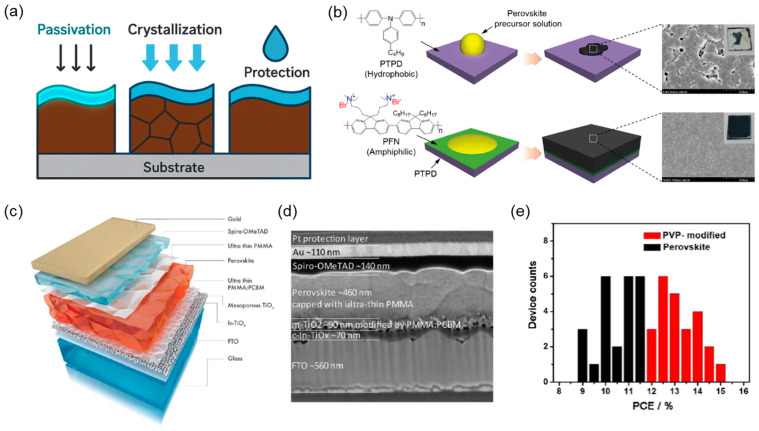
(**a**) Overview of polymer functions at PSC interfaces—passivation, crystallization, protection. (**b**) Schematic illustrations of perovskite film formation on organic HTLs with and without the interfacial compatibilizer, as well as corresponding photographs and SEM images. Reprinted with permission from Ref. [50] Copyright 2017, John Wiley and Sons. (**c**) Schematic of the device structure and (**d**) corresponding SEM cross-sectional image. Reprinted with permission from Ref. [56]. Copyright 2018, John Wiley and Sons. (**e**) PCE distributions of 24 devices for the devices with and without PVP modification. Adapted with permission from Ref. [58] Copyright 2017, John Wiley and Sons.

In addition to their established roles as charge transport layers, polymers serve a variety of interfacial functions in PSCs, such as compatibilization, surface passivation, and morphological control. The presence of interfacial polymer layers has demonstrably regulated film formation, aligned energy levels, and mitigated nonradiative losses at critical junctions. By modifying surface energy and eliciting chemical or physical interactions with the perovskite layer, polymers can suppress trap-assisted recombination, facilitate crystallization, and enhance long-term device stability (Figure 4a). A notable example of interfacial compatibilization was demonstrated using amphiphilic conjugated polyelectrolytes. The introduction of ultrathin poly[(9,9-bis(3′-(N,N-dimethylamino)propyl)-2,7-fluorene)-alt-2,7-(9,9-dioctylfluorene)] (PFN) layers on top of hydrophobic organic transport layers effectively addressed the issue of wettability mismatch with aqueous perovskite precursors, enabling uniform and pinhole-free film formation across large areas. The PFN-modified interface preserved charge transport while promoting better perovskite coverage and stability. Devices incorporating this strategy achieved PCEs exceeding 19% and retained performance under ambient conditions, as shown in Figure 4b [50].

Interface passivation using ultrathin polymer coatings has also been explored to minimize recombination losses. In particular, the combination of PFN at the hole interface with a thin LiF layer at the electron contact suppressed non-radiative recombination at both interfaces, thereby increasing the quasi-Fermi level splitting. This dual-passivation approach has been demonstrated to enable reproducible 1 cm^2^ devices with certified efficiencies up to 19.83%, while maintaining uniformity and stability over large areas [20]. The inherent trade-off between passivation and charge transport has also been addressed through nanostructured architectures. The patterning of TiO_2_ nanorods and the subsequent selective coating with an insulating polymer blend (PMMA:PCBM) resulted in the maintenance of charge-selective contact, whilst the remaining surface was passivated. This design allowed for localized extraction and extensive passivation simultaneously, resulting in an outstanding fill factor of 83.9% and a certified PCE of 21.6% [51].

From a fabrication perspective, scalable polymer deposition techniques have shown promise. The plasma polymerization of adamantane-based polymers yielded ultrathin, uniform coatings when applied directly to TiO_2_ without the use of solvents. The nanometer-scale interlayers improved perovskite coverage and reduced interfacial resistance, resulting in champion efficiencies over 19% and prolonged stability under ambient exposure for over 1000 h without encapsulation [52]. Direct surface passivation using insulating polymers such as PMMA has been particularly effective across various device configurations. The spin-coating of PMMA layers on CsPbI_2_Br perovskites has been shown to be an effective method of suppressing defect-assisted recombination and enhancing *V_oc_* [53]. Furthermore, the deployment of ultrathin PMMA films at both electron- and hole-selective interfaces consistently improved interfacial quality and device stability, as evidenced in wide band-gap PSCs and MAPbI_3_-based systems [54,55]. Bilateral application of PMMA continued the significant suppression of recombination losses at both contacts, pushing *V_oc_* to 1.22 V and yielding a PCE of 20.8%. The carbonyl groups in PMMA were key to this process, coordinating with Pb^2+^ ions to neutralize trap states (Figure 4c,d) [56].

Notably, both physical and chemical passivation mechanisms have emerged, and their efficacy depends strongly on the functional groups introduced at the perovskite surface. It has been demonstrated that the deposition of extremely dilute PMMA solutions via the spin-coating method results in the preferential accumulation of the solutions in grain boundaries and surface depressions. This phenomenon effectively isolates high-defect regions without forming continuous barriers. This approach resulted in enhanced fill factor and absolute PCE gains that surpassed 1% [57]. Beyond this physical isolation, carbonyl-bearing polymers (e.g., PMMA and related methacrylate/amide derivatives) provide Lewis-base carbonyl oxygen sites that can weakly coordinate undercoordinated Pb^2^⁺ and reduce surface trap density, complementing the geometrical isolation. Other Lewis base polymers, such as poly(4-vinylpyridine) (PVP), have shown analogous advantageous effects. Through the coordination of pyridyl nitrogen with undercoordinated Pb^2+^ ions, PVP treatment increased device performances as shown in Figure 4e [58]. Additionally, this PVP enhanced carrier lifetime and suppressed nonradiative pathways, delivering a champion *V_oc_* of 1.16 V and enhanced operational durability over 90 days [58,59]. In parallel, polymers such as PCDTBT have been applied as interfacial modifications, contributing to long-term stability and efficient charge extraction when deposited directly on the perovskite surface [60].

Solvent-free polymer coatings have also been realized using chemical vapor deposition polymerization of poly(p-xylylene), which offered a ~40 mV improvement in *V_oc_* and significantly enhanced moisture stability during long-term aging [61]. In a separate study, PVP coatings processed under ambient conditions were found to improve both film uniformity and resistance to moisture ingress. When applied as an ultrathin interfacial layer, PVP enabled PSCs to retain stable operation over 600 h and tolerate repeated mechanical deformation, demonstrating potential for flexible device platforms [62]. Passivation mechanisms can also be classified by their dominant functional chemistry as well as by physical or chemical characteristics. In particular, pyridyl groups (PVP-type) and carbonyl groups(ester/amide) show strong Lewis-base coordination to undercoordinated Pb^2^⁺ [58,59], while fluorinated side chains (–CF_3_, –C_n_F_2n+1_) provide hydrophobic surface passivation by lowering surface energy and suppressing moisture ingress; the latter can also introduce interfacial dipoles that assist band alignment when properly assembled. Complementary anchoring chemistries used in polymer or polymer-tethered layers (e.g., carboxylates and phosphonates for stronger binding, zwitterionic moieties for electrostatic screening) can be integrated into polymer backbones or side chains to simultaneously reduce defect density and improve environmental robustness. Hybrid strategies—such as zwitterionic interlayers, polymer blends, or SAM-tethered polymer brushes—thus combine electrostatic screening, dipole engineering, selective adhesion, and hydrophobic protection to stabilize large-area films under operational stress [57,58,59,60,61,62].

In summary, polymer-based interfacial layers—whether functioning via coordination bonding, surface energy tuning, or physical barrier formation—offer multifunctional, scalable solutions to reduce interfacial recombination and enhance device longevity. The tunability, ease of deposition, and compatibility with ambient or low-temperature processes of these materials make them essential design elements in both current and future PSC architectures.

## 6. Polymers as Transparent Electrodes for PSCs

**Figure 5:**
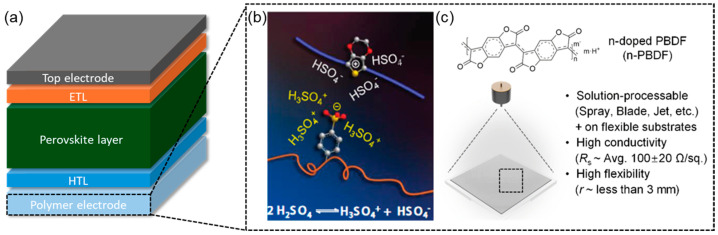
(**a**) Schematic PSC device structure with transparent conducting polymer as a bottom electrode. (**b**) PEDOT:PSS nanofibrils (**right**) via a charge-separated transition mechanism (**middle**) via a concentrated H_2_SO_4_ treatment. Adapted with permission from Ref. [73] Copyright 2014, John Wiley and Sons. (**c**) Schematic overview of the preparation of n-type conducting polymer (n-PBDF) film. Adapted with permission from Ref. [76] Copyright 2025, Royal Society of Chemistry.

Despite significant improvements in light absorption and charge transport layers in PSCs, the reliance on brittle and expensive transparent conductive oxides (TCOs) such as indium tin oxide (ITO) and costly noble metal electrodes like gold and silver remains a critical bottleneck to overcome for large-scale commercialization [63,64,65,66]. In recent years, conducting polymers have gained increasing attention as alternative electrodes for PSCs, providing a combination of flexibility, transparency, and processability through low-temperature solution techniques. When polymers are used as transparent electrodes in PSCs, as shown in Figure 5a, their performance is primarily assessed through three critical parameters: optical transmittance, sheet resistance, and work function alignment [67,68]. High optical transmittance (typically >85% in the visible range) ensures efficient photon delivery to the photoactive layer, while low sheet resistance (ideally <100 Ω/sq) is essential for efficient lateral charge transport, which is evaluated using a figure of merit to quantify the balance between optical transmittance and electrical conductivity [68].

Especially for the scale-up of PSCs, the lower the sheet resistance, the greater the minimization of energy loss during lateral charge transport. Notably, conducting polymers offer tunable work functions that facilitate effective energy level alignment with adjacent charge transport layers, minimizing interfacial losses [67]. Additionally, their flexibility and compatibility with low-temperature processing are key considerations, especially for flexible or roll-to-roll processed PSCs [68]. Recent advances in polymer composites, such as those blended with silver nanowires, carbon nanotubes, or graphene, have enabled simultaneous improvements in conductivity and transparency, approaching the performance benchmarks of traditional ITO electrodes while offering superior mechanical resilience, which are well reviewed in other papers and will not be discussed in this review [69,70,71]. Here, we mainly focus on only polymer-based electrodes for PSCs [67,72].

Conducting polymers such as PEDOT:PSS, PANI, polypyrrole (PPy), and newly developed n-type polymers such as poly(benzodifurandione) (n-PBDF) have demonstrated their potential to function as transparent electrodes for PSCs. In particular, PEDOT:PSS has been extensively studied since the development of OSCs, as its conductivity can be significantly enhanced through acid doping or solvent doping (Figure 5b) [73]. For instance, Zhu et al. demonstrated the feasibility of FAI-treated PEDOT:PSS as a transparent electrode for flexible PSCs, where FAI refers to formamidinium iodide [74]. The FAI-treated PEDOT:PSS electrode exhibited high optical transparency (over 85% transmittance from 350 nm to 550 nm and around 75% transmittance from 550 nm to 900 nm) and moderate electrical conductivity (1562 ± 36 S cm^−1^), enabling its application in both rigid and flexible PSC architectures. Devices fabricated on the polymer/glass substrate achieved a PCE of 13.36%, compared to 16.60% on conventional ITO/glass, while flexible devices using polymer-coated PET substrates reached a PCE of 10.16%. The reduced efficiency was attributed primarily to the lower conductivity of the polymer electrode and interfacial charge recombination. Despite these limitations, the work highlights a scalable, low-temperature processing route for transparent and flexible electrodes, suggesting future potential through conductivity optimization and interface engineering.

Moreover, by introducing fluorosurfactant to regulate the phase separation in PEDOT:PSS formulation, Hu et al. developed a mechanically robust PEDOT:PSS electrode to bridge the performance gap between flexible and rigid PSCs [75]. The resulting polymer network achieved exceptional electrical conductivity (>4000 S cm^−1^), high optical transmittance (>80% from 400 nm to 900 nm), and strong mechanical endurance. Flexible PSCs fabricated with this electrode exhibited high PCEs of 19.0% (0.1 cm^2^) and 10.9% (25 cm^2^), closely matching rigid device performance. Additionally, when employed as the top electrode in semi-transparent PSCs, the device achieved 12.5% PCE with an average visible transmittance of 30.6%. The PSCs demonstrated excellent mechanical stability, retaining over 80–90% of their initial efficiencies after 5000 bending cycles at a 3 mm bending radius, validating the electrode’s suitability for wearable and rollable photovoltaic applications.

Unlike p-type conducting polymers such as PEDOT:PSS, which are limited to inverted device structures, n-doped conducting polymers enable the fabrication of conventional planar PSC architectures. Kumar et al. reported the development of transparent conducting polymer electrodes based on n-doped conducting polymer (n-PBDF), offering a viable alternative to conventional ITO in PSCs (Figure 5c) [76]. The polymer electrodes with the optimal thickness range of 65–75 nm (90.9–78.8 Ohm sq^−1^) provide a suitable balance between optical transparency and electrical conductivity. Integration of n-PBDF into both rigid and flexible PSCs resulted in PCEs of 12.70% and 11.23%, respectively, with minimal impact on perovskite film quality, morphology, or optoelectronic properties. This work confirms the potential of n-type conducting polymers for developing ITO-free, flexible, and low-cost PSCs, addressing a critical bottleneck in transparent electrode technology.

Despite recent progress, the widespread adoption of conducting polymers as transparent electrodes in PSCs still faces several critical challenges: further improving electrical conductivity and optical transparency by advanced doping strategies and nano-structuring approaches—such as incorporating metal grid, metal nanowires or carbon-based nanomaterials; ensuring mechanical robustness and environmental stability by developing cross-linked or self-healing polymer networks; the work function tunability of n-type and p-type conducting polymers to enable better energy-level alignment with transport layers in both conventional and inverted PSC architectures; scalable, low-temperature, and solvent-compatible processing techniques are essential to ensure compatibility with large-area, roll-to-roll manufacturing. Addressing these limitations could accelerate the commercial viability of polymer-based transparent electrodes in next-generation, flexible, and light weight photovoltaic technologies.

## 7. Polymers as Encapsulation Layers for PSCs

One of the most pressing challenges impeding the commercial deployment of PSCs is poor long-term environmental stability. Perovskite materials are highly sensitive to moisture, oxygen, heat, and ultraviolet (UV) light, which induce rapid degradation, particularly under ambient and outdoor conditions [77,78,79,80]. To address this, advanced encapsulation strategies are crucial, and polymeric materials have recently emerged as highly effective candidates due to their flexibility, processability, and tunable barrier properties [81,82,83,84,85,86,87]. The fundamental role of a polymer encapsulant is to isolate the photoactive layers from environmental stressors such as moisture, oxygen, dust, and UV radiation. Thus, effective polymer encapsulants must form a conformal and pinhole-free film that prevents the diffusion of ambient air into the device while maintaining optical transparency and mechanical compliance. Moreover, certain polymers, such as fluoropolymers, polyurethane (PU), and poly(methyl methacrylate) (PMMA), can be engineered to exhibit low water vapor transmission rate and low oxygen transmission rate (OTR), ideally below 10^−5^ g·m^−2^·day^−1^ and 10^−3^ cm^3^·m^−2^·day^−1^·atm^−1^, respectively, which makes them suitable for both rigid and flexible PSC architectures. Advanced polymer formulations may also incorporate self-healing, cross-linking, or lead-sequestration functionalities, further enhancing long-term durability and environmental safety. Therefore, the strategic integration of polymer encapsulants plays a critical role in advancing PSCs toward real-world stability and commercialization.

**Figure 6:**
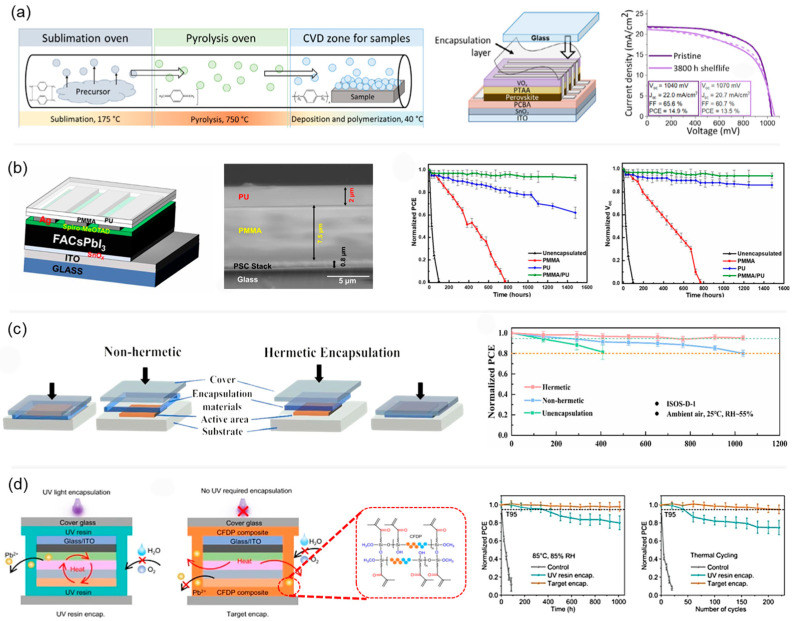
(**a**) Parylene-N encapsulation of MAPbI_3_ PSCs via CVD improves shelf-life stability without compromising device performance. Adapted with permission from Ref. [88] Copyright 2025, John Wiley and Sons. (**b**) Cross-sectional structure and long-term operational stability of FACsPbI_3_ PSCs with different encapsulation strategies. The bilayer PMMA/PU structure significantly retains both PCE and *V_oc_* over 1500 h under ambient storage (25 °C, RH ~50%, dark), compared to single-layer PMMA, PU, and unencapsulated devices. Adapted with permission from Ref. [89] Copyright 2024, Royal Society of Chemistry. (**c**) Comparison of hermetic and non-hermetic encapsulation on PSCs under heat and light stress, showing improved morphology retention, structural stability, and device performance with hermetic sealing. Adapted with permission from Ref. [90] Copyright 2025, American Chemical Society. (**d**) Schematic illustration of UV resin-encapsulated, and CFDP-based target-encapsulated PSCs, including the molecular structure of the CFDP composite, and their J–V characteristics and long-term stability under damp heat (85 °C, 85% RH) and thermal cycling (−40 °C to 85 °C) conditions. The molecular structure of a self-crosslinked fluorosilicone polymer gel is in Red dotted lines. Adapted with permission from Ref. [91] Copyright 2023, Springer Nature.

Among the latest advancements, Zagorovskaia et al. demonstrated the effectiveness of poly-para-xylylene (parylene N) as a robust encapsulation material, providing hydrophobic characteristics and excellent barrier performance (Figure 6a) [88]. PSCs encapsulated with parylene N and covered glass exhibited impressive operational stability exceeding 3800 h in dark ambient conditions, retaining 92% of their initial PCE. Furthermore, the polymer’s compatibility with low-temperature deposition processes makes it suitable for flexible substrates and tandem devices. Interestingly, Raman et al. reported a simple and scalable bilayer polymer encapsulation strategy to enhance the environmental stability of PSCs [89]. By combining a hydrophilic PMMA layer with a hydrophobic PU layer, the authors developed a protective barrier that effectively blocks moisture and oxygen infiltration without damaging the device structure (Figure 6b). Under high humidity conditions (80 ± 5% RH), the encapsulated PSCs showed no degradation for over 28 days and retained more than 92% of their initial PCE after 1500 h. In contrast, unencapsulated devices degraded rapidly within 48 h. The approach demonstrates the strong potential of polymer encapsulants for application in flexible and scalable optoelectronic devices.

Yang et al. investigated the role of hermetic encapsulation in enhancing the operational stability of PSCs, as shown in Figure 6c [90]. Unsealed or poorly sealed devices exhibited rapid degradation under continuous 1-sun illumination, showing a PCE drop of 20% within 300 h, accompanied by morphological changes such as the formation of coral-like crystal structures due to a decomposition–nucleation–regrowth process. In contrast, PSCs encapsulated with pressure-applied barrier films retained more than 90% of their initial PCE after 1000 h of operation. The hermetically sealed devices also suppressed ion migration and PbI_2_ reformation, key factors driving long-term degradation. This study emphasizes that encapsulation is not merely a protective covering but a functional layer essential for preserving both the morphological integrity and optoelectronic performance of PSCs, particularly under heat, light, and moisture stress. Unlike traditional encapsulants, the fluorosilicone gel offers simplified processing, effective thermal dissipation, and excellent suppression of lead leakage. Li et al. developed a room-temperature, nondestructive encapsulation method using a self-crosslinked fluorosilicone polymer gel to enhance the environmental and thermal stability of PSCs (Figure 6d) [91]. Devices encapsulated using this method retained 98% of their PCE after 1000 h under damp heat (85 °C/85% RH) and 95% after 220 thermal cycles, fully meeting the IEC 61,215 stability criteria. Additionally, the encapsulated cells achieved > 98% lead containment efficiency in immersion and rain exposure tests. This encapsulation strategy provides a universal and scalable path toward stable, sustainable, and environmentally safe perovskite photovoltaics. Table 1 summarizes the details of the encapsulated device performance.

Taken together, these studies reflect a significant shift toward multifunctional polymer encapsulation technologies that serve not only as protective barriers but also as performance enhancers. The future of PSC encapsulation will likely involve hybrid strategies combining self-healing, UV filtering, moisture barrier, and flexible properties into a single polymeric system—paving the way for efficient, durable, and scalable perovskite solar modules.

**Table 1:** The summary of the encapsulation strategies and the encapsulated device performance.

Device Structure	Encapsulation Materials	Stability Test Condition	Performance	Ref.
Glass/ITO/SnO_2_/PCBA/MAPbI_3_/PTAA/VO_x_/Ag	Parylene-N + cover glass	Dark ambient (22–26 °C, 30–40% RH)	PCE retained 92% of initial value after 3800 h	[88]
Glass/ITO/SnOx/FA_0.9_Cs_0.1_PbI_3_/spiro-OMeTAD/Ag	PMMA/PU bilayer	Dark ambient (25 ± 3 °C, RH 80 ± 5%)	PCE retained 92% after 1500 h	[89]
FTO/SnO_2_/Cs_0_._05_(FA_0.85_MA_0.15_)_0.95_Pb(Br_0.15_I_0.85_)_3_/Spiro-OMeTAD/Au	hermetic (Surlyn + glass)	Ambient air (25 °C, RH 55%), dark storage	PCE retained 95.3% after 1036 h	[90]
Glass/ITO/NiO_x_/Perovskite/PCBM/C_60_/BCP/Au+Cr	CFDP + cover glass	Thermal cycling (−40 °C to 85 °C, 220 cycles)	Retained 95% of initial PCE	[91]
Glass/FTO/c-TiO_2_/mp-TiO_2_/Cs_0.10_FA_0.90_Pb(I_0.83_Br_0.17_)_3_/HTL/Ag	Laser-assisted glass frit	70 thermal cycles (−40 °C to 85 °C) + 50 h damp heat (85 °C, 85% RH)	Retained 97% of initial PCE after 50 h	[92]
Glass/ITO/NiO_x_/Cs_0.17_FA_0.83_Pb(I_0.83_Br_0.17_)_3_/PC60BM/SnO_2_/ZnSnO_2_/ITO/Ag	Ethylene vinyl acetate (EVA) polymer or ionomer Surlyn 5400 + glass	thermal cycling 200 cycles (−40 °C to 85 °C)	Retained 90% of initial PCE	[93]
Glass/ITO/NiO/Cs_0.17_FA_0.83_Pb(Br_0.17_I_0.83_)_3_/LiF/PCBM/SnO_2_/ZTO/ITO/Ag	EVA + glass, butyl rubber (edge seal)	damp heat test: 85 °C, 85% RH, 1000 h	PCE maintains over 90% after 1000 h	[94]

## 8. Polymers as Anti-Reflection Layers for PSCs

Although the reported PCE of PSCs has surpassed 27%, minimizing optical and recombination losses remains essential to approach the fundamental theoretical limit of over 30% [95]. Thus, light management is a critical component in the design of high-efficiency PSCs [96,97,98]. Due to inherent optical losses from surface reflection, the integration of anti-reflection (AR) coatings has proven effective in improving light harvesting, particularly across the visible spectrum. Polymers have recently emerged as versatile AR materials due to their tunable refractive indices, solution processability, and mechanical flexibility, all of which are essential for next-generation solar technologies, including flexible and tandem architectures [99,100].

The principle of AR is fundamentally based on the destructive interference of reflected light waves at material interfaces. When a thin dielectric layer with an appropriate refractive index (n) is coated onto a substrate, partial reflections from the air/film and film/substrate boundaries interfere destructively if the optical path difference equals half the wavelength (λ/2). For maximum cancellation, the n of the AR layer is ideally chosen as the geometric mean of the refractive indices of air and the substrate ($n_{\mathrm{AR}}=\sqrt{n_{\mathrm{air}}\;n_{\mathrm{substrate}}}$), and the film thickness is typically a quarter of the target wavelength (λ/4n) [99,101]. This approach minimizes reflection and enhances transmission within the photoactive layers of optoelectronic devices, resulting in improved photon harvesting by reducing Fresnel losses at the air/glass or air/encapsulation interfaces, leading to enhanced short-circuit current density (*J_sc_*) and thereby overall PCE.

**Figure 7:**
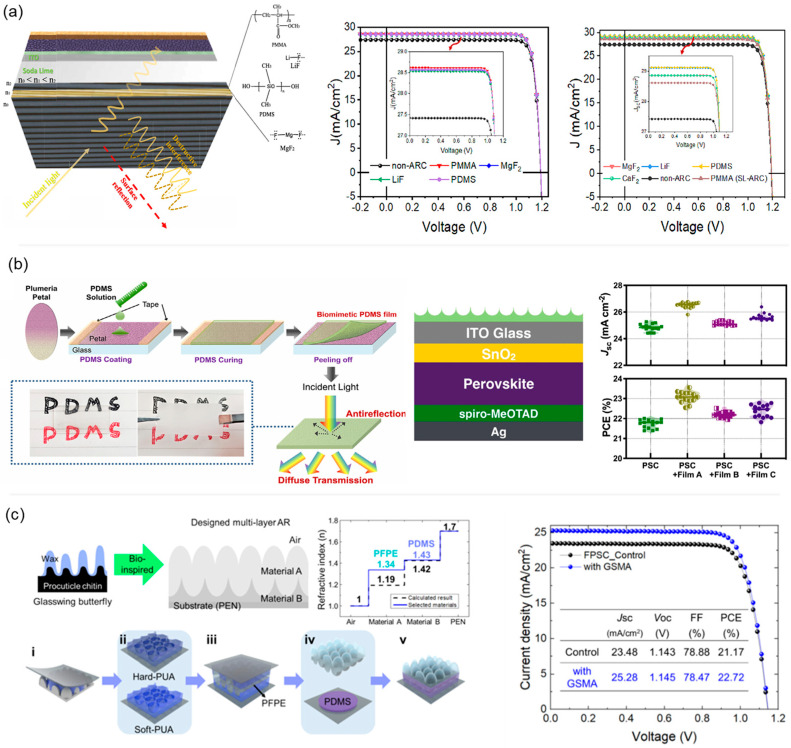
(**a**) Device architecture of PSCs incorporating a PMMA/PDMS-based single-layer anti-reflective coating (SL-ARC and DL-ARC) and their chemical structures, along with the simulated J-V curves with SL-ARC (left) and DL-ARC (right). Adapted with permission from Ref. [102] Copyright 2024, Elsevier. (**b**) Fabrication process and surface morphology of biomimetic PDMS films, device architecture of PDMS-integrated PSCs, and corresponding photovoltaic parameters (*J_sc_*, PCE) for different PDMS film types. Adapted with permission from Ref. [103] Copyright 2024, John Wiley and Sons. (**c**) Design and fabrication process of GSMA film with actual wing structure of the glasswing butterfly, showing improved photovoltaic performance, especially *J_sc_* compared to the control device. Adapted with permission from Ref. [104]. Copyright 2025, Elsevier.

Almuqoddas et al. theoretically simulated the effect of single-layer and double-layer AR coatings (SL-ARC and DL-ARC) on the performance of an inverted p–i–n PSC (Figure 7a) [102]. Initially, by optimizing the photoactive layer thickness, charge carrier mobility, and recombination rates, the maximum attainable efficiency under ideal internal conditions was predicted to be 28.4%. However, this value still falls short of the theoretical limit due to optical losses caused by surface reflection. To address this, SL-ARC and DL-ARC were introduced. Application of a 70 nm thick PMMA-based SL-ARC increased the PCE to 29.7%, while a dual-layer PMMA/polydimethylsiloxane (PDMS) DL-ARC further enhanced the PCE beyond 30%, effectively reaching the theoretical efficiency limit. These simulated results clearly show the necessity of AR coatings for high-performance PSCs.

Interestingly, Ma et al. developed a biomimetic AR film based on PDMS to enhance the optical management of PSCs [103]. Inspired by the hierarchical surface structures of plumeria flower petals, the authors replicated micro- and nano-patterns onto PDMS substrates using a soft-molding technique, as illustrated in Figure 7b. These films with specific patterns exhibited strong light-scattering capabilities, resulting in significantly reduced surface reflectance and broadband transmission enhancement in the visible range. Integration of the biomimetic AR film led to a PCE of 23.56%, with a notable *J_sc_* of 26.54 mA cm^−2^, corresponding to an average efficiency improvement of 6.17%. The performance enhancement was validated under various lighting conditions, including AM 1.5G solar irradiation at different angles and light-emitting diode (LED) illumination of varying intensities. Optical simulations further confirmed the correlation between microstructure geometry and light-trapping efficiency. This study highlights the potential of nature-inspired polymer encapsulation films in advancing PSC performance through scalable, low-cost AR strategies.

In addition, Choi et al. proposed a novel anti-reflective film that was inspired by the nano-micro hierarchical structure of glasswing butterfly wings, fabricating a multilayer AR coating with gradient refractive index properties (Figure 7c) [104]. The resulting film minimized front-side optical reflection and simultaneously provided mechanical resilience compatible with flexible substrates. When applied to flexible PSCs, the AR film exhibited a substantial enhancement in light absorption and device stability under repetitive bending stress. These studies demonstrate a promising route toward high-efficiency, flexible PSCs via the integration of biomimetic AR functionality with structural robustness.

Polymer-based AR coatings represent a rapidly developing frontier in PSC technology. Their multifunctionality, structural tunability, and compatibility with low-cost fabrication methods make them promising candidates for both high-performance and scalable photovoltaic applications. Ongoing research is expected to further integrate these AR layers with encapsulation and passivation functionalities.

## 9. Polymers as Interconnecting Layers for Perovskite Tandem Cells

**Figure 8:**
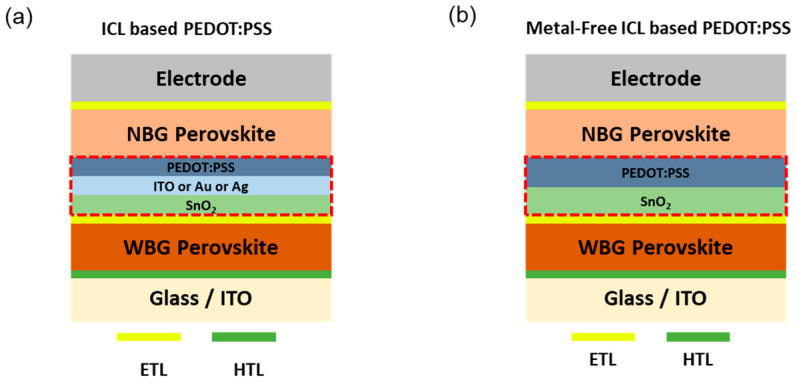
Schematic device architectures of perovskite tandem solar cells employing PEDOT:PSS-based interconnecting layers (ICL) (**a**) with and (**b**) without metal layers. Red boxes are recombination layers.

The use of perovskite/perovskite tandem solar cells has emerged as a promising strategy to surpass the theoretical efficiency limit of single-junction perovskite devices. By stacking wide-bandgap and narrow-bandgap perovskite absorbers, tandem structures enable a broader solar spectrum to be harvested. However, efficient charge recombination, energy level alignment, and interface passivation between the sub-cells are essential to achieving high device performance and long-term operational stability. Notably, polymer-based interconnecting layers (ICLs) have attracted significant attention as multifunctional interface engineering materials due to their solution processability, tunable optoelectronic properties, and mechanical flexibility [34,37]. Yang et al. introduced SnO_2_/ITO/PEDOT:PSS as ICL in all-perovskite tandem solar cells by showing a monolithic all-perovskite tandem cell PCE of 22.7% (Figure 8a) [105]. A crucial enabler of the tandem architecture was the use of a PEDOT:PSS-based ICL, which facilitated efficient hole recombination and preserved the optical and electrical integrity between the top and bottom sub-cells. The PEDOT:PSS ICL’s conformal coverage, transparency, and energetic alignment were key to minimizing series resistance and maximizing tandem performance.

Likewise, Abdollahi Nejand et al. successfully demonstrated scalable two-terminal all-perovskite tandem solar modules implementing an ICL architecture using SnO_x_/ITO or Au/PEDOT:PSS (Figure 8a), where PEDOT:PSS served as the hole-transport and recombination layer between the wide-bandgap top and narrow-bandgap bottom sub-cells [36]. The resulting tandem module achieved an aperture-area PCE of 19.1% over a large area of 12.25 cm^2^ with a geometric fill factor of 94.7%. Additionally, the PEDOT:PSS ICL was compatible with laser scribing and did not compromise module uniformity, as confirmed by electroluminescence and laser-beam-induced current (LBIC) mapping. Lin et al. developed high-efficiency monolithic all-perovskite tandem solar cells by addressing the key instability issue in tin-based narrow-bandgap perovskites: the oxidation of Sn(II) to Sn(IV) [38]. They introduced an ICL architecture of ALD-SnO_2_/Au/PEDOT:PSS in all perovskite tandem solar cells, achieving a certified small-area PCE of 24.8% (0.049 cm^2^) and 22.1% for larger area (1.05 cm^2^) devices. The PEDOT:PSS layer in ICL served as an efficient hole recombination layer, facilitating energy level alignment, maintaining optical transparency, and allowing low-temperature processing compatible with the perovskite layers. Its inclusion was critical in ensuring minimal series resistance and in maintaining the structural and operational integrity of the tandem stack, which retained over 90% of its initial performance after 463 h under 1-sun continuous illumination at the maximum power point. Consequently, these studies elucidate the critical role of conductive polymers like PEDOT:PSS in achieving scalable, efficient, and low-cost all-perovskite tandem photovoltaics.

Xu et al. developed a metal-free ICL for monolithic perovskite/organic tandem solar cells, consisting of atomic layer-deposited tin oxide (SnO_2_) combined with diluted PEDOT:PSS (Figure 8b) [35]. The authors fully eliminated the need for parasitically absorbing metallic recombination layers typically used in tandem architectures. This configuration provided a highly transparent, chemically stable, and energetically favorable interface between the perovskite and organic sub-cells. Importantly, the all-metal-free tandem device achieved a comparable PCE to the metal-based counterpart while exhibiting significantly enhanced photostability under prolonged light exposure. Outdoor stability tests over 2 weeks of real sunlight exposure showed that the monolithic tandem device outperformed both single-junction perovskite and OSCs in terms of retained PCE and thermal/photo durability, highlighting the practical advantage of this metal-free interface strategy for scalable tandem photovoltaics.

For scalable production of PSCs, a roll-to-roll printable polymer interlayer system with uniform film formation and good interfacial adhesion needs to be developed. This technology paves the way for the commercial viability of large-area tandem modules. Overall, polymers in interconnecting layers play a pivotal role in enhancing the optoelectronic coupling and mechanical/chemical stability of perovskite/perovskite tandem cells. The next generation of tandem photovoltaics will surely depend on further development of multifunctional, scalable polymer interconnecting layers tailored for both interface and device-wide performance optimization.

## 10. Challenges and Considerations for Large-Area Module Integration

Integrating polymer functional layers into large-area PSC modules presents significant hurdles, primarily in controlling film morphology and ensuring long-term device stability. Transitioning from lab-scale spin-coating to scalable methods such as slot-die or blade coating is essential but difficult, as altered fluid dynamics and drying kinetics often induce morphological defects. These issues, including non-uniform thickness, pinholes, and the coffee-ring effect, create shunt pathways that critically degrade the device’s fill factor and efficiency. Key research strategies to mitigate these problems involve optimizing ink formulations through solvent engineering, precisely controlling processing parameters, and designing new polymers tailored for high-throughput deposition. Stability issues become even more pronounced at the module level. Large-area devices are prone to heat accumulation and hot spots that can thermally degrade the polymer layers. In flexible roll-to-roll devices, mechanical stress can cause cracking or delamination, leading to electrical failure. Furthermore, as the difficulty of achieving complete encapsulation of large modules increases, the risk of moisture and oxygen ingress rises, accelerating chemical degradation at sensitive internal interfaces. Therefore, resolving the interrelated challenges of morphology and stability during the scale-up process remains a key challenge for the commercialization of PSCs with polymer functional layers.

## 11. Computational Modeling in Polymer Design

Beyond traditional experimental synthesis and characterization, the integration of computational modeling is emerging as a powerful strategy to accelerate the discovery of novel polymer functional layers. Theoretical methods offer profound insights into the properties that govern device performance, enabling a more rational design approach. For instance, Density Functional Theory (DFT) calculations can accurately predict the frontier molecular orbital energies (HOMO/LUMO) of yet-to-be-synthesized polymer candidates, allowing for an initial screening based on the prerequisite energy level alignment with the perovskite active layer.

Furthermore, as highlighted in recent computational studies, theoretical models can effectively simulate charge transport phenomena and thermal behavior at complex interfaces, including those between polymers and perovskites [106]. Molecular Dynamics (MD) simulations complement these electronic structure calculations by predicting polymer chain packing, bulk morphology, and the dynamic interactions at the polymer–perovskite junction, which are critical for both charge mobility and long-term stability. The significance of this approach lies in its potential for high-throughput virtual screening, where vast libraries of candidate molecules can be computationally evaluated in silico. This allows researchers to prioritize the most promising candidates for synthesis, creating an efficient feedback loop between theory and experimentation and significantly accelerating the development of next-generation materials for PSCs.

## 12. Summary

In summary, we reviewed the applications of polymers as functional layers and analyzed the commercialization challenges of PSCs. As charge transport layers, such as ETLs and HTLs, various polymers have appropriate energy levels and mobility for both normal and inverted structured PSCs. In particular, broadly utilized materials such as PTAA demonstrate performance comparable to that of SAMs or other small molecules that were originally developed for OLED applications before being adapted for use in other devices. In addition to the charge transport functional layers, polymers have been broadly applied as an interfacial layer for better wettability and the passivation of perovskite. They have also been applied as an encapsulation layer to prevent oxygen or moisture penetration. Conducting polymers such as PEDOT:PSS, PANI, and PPy have been adopted as electrodes for semitransparent and flexible PSCs. Thus far, polymers originally utilized in organic solar cells (OSCs), OLEDs, and DSSCs have been adapted and further optimized for integration into PSCs.

For more efficient and stable device operation, the advancement of novel dopant technologies or the development of new materials is likely required. In particular, under outdoor operating conditions, polymer functional layers in PSCs must withstand multiple stress factors, including UV exposure, thermal cycling, and ambient humidity for competitive outdoor performance. Polymer layers should maintain over 90% of their initial functionality after 1000 h of damp heat testing (85 °C/85% RH) and resist UV-induced degradation (e.g., under 1-sun AM 1.5G for 500–1000 h) without significant chemical or morphological changes. Glass transition temperatures (T_g_) above 150 °C, UV absorption thresholds above 350 nm, and water vapor transmission rates (WVTR) < 10^−5^ g·m^−2^·day^−1^ are desirable targets for encapsulating or barrier-type polymer layers. Advances in crosslinked networks, fluorinated backbones, or UV-stabilized side chains help polymers close the stability gap with inorganics, and hybrid designs incorporating organic–inorganic nanocomposites may offer the best of both worlds. We hope this review will accelerate the development of polymer functional layers that can contribute to the commercialization of PSCs.

## Data Availability

No new data were created or analyzed in this study.

## Abbreviations

The following abbreviations are used in this manuscript:

PSC	Perovskite solar cells
PCE	Power conversion efficiency
*J_sc_*	Short-circuit current density
*V_oc_*	Open-circuit voltage
DSSC	Dye-sensitized solar cells
HTL	Hole transport layer
ETL	Electron transport layer
Spiro-OMeTAD	2,2′,7,7′-Tetrakis(N,N-di-p-methoxyphenylamine)-9,9′-spirobifluorene
PEDOT:PSS	Poly(3,4-ethylenedioxythiophene):poly(styrenesulfonate)
PTAA	Poly[bis(4-phenyl)(2,4,6-trimethylphenyl)amine]
OSC	Organic solar cells
PANI	Polyaniline
PMMA	Poly(methyl methacrylate)
AR	Anti-reflection
PDMS	Polydimethylsiloxane
LED	Light-emitting diode
ICL	Interconnecting layer
ALD	Atomic layer deposition
P3HT	Poly(3-hexylthiophene)
PEI	Polyethylenimine
PVP	Poly(4-vinylpyridine)
PAA	Polyallylamine
PU	Polyurethane
n-PBDF	Poly(benzodifurandione)
